# Evidence-based pragmatic guidelines for the follow-up of testicular cancer: optimising the detection of relapse

**DOI:** 10.1038/sj.bjc.6604280

**Published:** 2008-06-10

**Authors:** N J van As, D C Gilbert, J Money-Kyrle, D Bloomfield, S Beesley, D P Dearnaley, A Horwich, R A Huddart

**Affiliations:** 1 Royal Marsden Hospital and The Institute of Cancer Research, Sutton, UK; 2 Royal Surrey County Hospital, Guildford, UK; 3 Royal Sussex County Hospital, Brighton, UK; 4 Kent Cancer Centre, Maidstone, UK

## Abstract

Testicular germ cell tumours (TGCTs) are the most common cause of cancer in men between the ages of 15 and 40 years, and, overall, the majority of patients should expect to be cured. The European Germ Cell Cancer Consensus Group has provided clear guidelines for the primary treatment of both seminoma and nonseminomatous germ cell tumours. There is, however, no international consensus on how best to follow patients after their initial management. This must promptly and reliably identify relapses without causing further harm. The standardising of follow-up would result in optimising risk-benefit ratios for individual patients, while ensuring economic use of resources. We have identified the seven common scenarios in managing seminomas and nonseminomas of the various stages and discuss the pertinent issues around relapse and follow-up. We review the available literature and present our comprehensive TGCT follow-up guidelines. Our protocols provide a pragmatic, easily accessible user-friendly basis for other centres to use or to adapt to suit their needs. Furthermore, this should enable future trials to address specific issues around follow-up giving meaningful and useful results.

## 

### Background

Testicular germ cell tumours (TGCTs) are uncommon malignancies but the most common cause of cancer in men between the ages of 15 and 40 years. The peak incidence for nonseminomatous germ cell tumour (NSGCT) is between 20 and 30 years of age, and for seminoma between 30 and 40 years. In the United Kingdom, the incidence rate is only 1 : 100 000 men per year with a lifetime risk of developing a TGCT of 1 in 400 and 1900 new cases per year (Horwich, 2002). There has, however, been a steady increase in the incidence of TGCTs in European countries in the last two decades (Bergstrom *et al*, 1996). The reasons for this increasing incidence and the aetiology of TGCTs remain unknown. The European Germ Cell Cancer Consensus Group has provided clear guidelines for the primary treatment of both seminoma tumour and NSGCT (Schmoll *et al*, 2004). There is, however, a lack of clear consensus on how to follow patients after primary treatment, and a number of issues dictate that follow-up should be carefully thought out and rigorously adhered to. Here, we discuss these factors as they pertain to male germ cell tumour (GCT) practice and describe our recently developed protocols.

### Rational for follow-up

#### Detecting relapse

In general, detecting relapse is the major reason for maintaining follow-up and is the main focus of this review. The management of testicular cancer has been a major oncological success story, and provides a model for the management of curative solid tumours (Horwich *et al*, 2006). The use of platinum-based chemotherapy schedules has resulted in high cure rates for all stages of the disease (International Germ Cell Cancer Collaborative Group, 1997). The fact that the majority of young men treated for testicular cancer have a durable response to primary treatment has resulted in the accumulation of significant data on both the patterns of relapse and the late effects of treatment. Lifelong cure rates are high but there remains a tangible risk of relapse. These relapses may be salvageable with a combination of further chemotherapy and surgery; recent studies suggest that around 50% of patients who relapse after primary treatment will be cured, depending on the pattern of relapse and the stage at detection (Fossa *et al*, 1999b; Huddart and Birtle, 2005). It is also recognised that late relapses (occurring greater than 2 years after complete remission) have a greater propensity for chemoresistance and confer a worse prognosis (Shahidi *et al*, 2002; Ronnen *et al*, 2005). The fact that relapses are discernable through the use of serum tumour markers and/or radiological imaging confers a responsibility for their prompt detection. However, false-negative computerised tomography (CT) examinations can occur owing to the inability of this modality to detect foci of disease in normal-sized nodes or to differentiate benign and malignant enlargement. It has been shown that by using 10–15 mm as the upper limit of normal, up to 44% of scans were false negative (Thomas *et al*, 1981; Richie *et al*, 1982; Rowland *et al*, 1982). For practical purposes then, a cutoff of 10 mm is used treating those measuring between 8 and 10 mm as suspicious. These measurements should be taken in the overall context of the patient (risk of disease, markers and tumour laterality) (Dalal *et al*, 2006). Tumour markers (*β*HCG and/or AFP) are elevated at relapse in about 2/3 of NSGCT and approximately 1/3 of seminomas. While LDH is vital in prognostication of metastatic disease and thus should be routinely included, its use in detecting relapse is questionable (Ackers and Rustin, 2006; Venkitaraman *et al*, 2007). Although markers may act as useful flags for relapse, they do not remove the need for clinical and imaging assessment in view of the rate of marker-negative relapse.

#### Detection of second primary cancer

A separate issue is that of metachronous, contralateral primary testicular tumours. Although a number of studies have reported an increased incidence, in the absence of specific risk factors (testicular maldescent, infertility, atrophy, young age at first presentation and microlithiasis; Harland *et al*, 1998; Holm *et al*, 2003) this risk is still low with a 15-year cumulative incidence of just 1.9% (Fossa *et al*, 2005). Apart from being encouraged to perform routine self-examination, we would not, therefore, routinely perform contralateral biopsies as part of our follow-up. The major risk factor for second primary cancer is thought to be testicular atrophy (Harland *et al*, 1998). Thus, in patients with a testicular volume of <12 ml, we would discuss the role of testicular biopsy to detect carcinoma *in situ* (before or at least 2 years post chemotherapy). If detected, carcinoma *in situ* is managed appropriately (Hoei-Hansen *et al*, 2004).

#### Late effects: physical

A third potential reason for undertaking follow-up is to assess, monitor and manage late toxicity of treatment. In addition to the well-recognised acute side effects of chemotherapy the significance of late side effects have become increasingly appreciated. Long-term survivors of testicular cancer may display an increased risk of cardiovascular disease (Huddart *et al*, 2003; Huddart and Birtle, 2005; van den Belt-Dusebout *et al*, 2006) and second malignancy (Travis *et al*, 2005; Raghavan *et al*, 2006). Other problems identified include long-term sensory neuropathy, Raynaud's phenomenon (Fossa, 2004) and effects on fertility (Brydoy *et al*, 2005; Huddart *et al*, 2005). Development of ‘metabolic syndrome’ and other long-term endocrine disturbance is also recognised (Nuver *et al*, 2005; Horwich *et al*, 2006). To date, detection and management of toxicity have been a minor component of most oncologists practice, and, in the main, this aspect has, by default, been delegated to primary care. Raghavan has made some recommendations but the evidence base for making recommendations in this area is limited. Detailed discussion of these issues is beyond the scope of this review, although we have made some rudimentary suggestions for late effect monitoring.

#### Late effects: psychological

A proportion of testicular cancer patients are psychologically vulnerable and find the diagnosis of testicular cancer traumatic. Subsequent treatment may significantly affect sexual function, cause anxiety, disrupt ability to work and reduce overall quality of life (Fleer *et al*, 2006; Dahl *et al*, 2007; Tuinman *et al*, 2007). Follow-up can help support such patients and help to rehabilitate so that they return to normal life.

#### Research

Research is rarely the major goal of follow-up, but the long-term follow-up of patients and collection of data within prospective trials and cohort-based databases have undoubtedly contributed significantly to understanding testicular cancer and treatment outcomes.

### Risks of radiological intervention

Repeated use of imaging techniques that utilise ionising radiation (specifically chest radiographs and CT scanning of the thorax, abdomen and pelvis) is associated with a measurable risk of causing second malignancies. These risks must be outweighed by demonstrable benefit to patients (Hoffman *et al*, 1989; Boice *et al*, 1991; Brenner, 2004; Ronckers *et al*, 2005). Thoracic CT requires a radiation dose equivalent to 400 chest radiographs (8 *vs* 0.02 mSv), increasing the exposure to 20 mSv if the abdomen is included. It has been suggested that this results in a 1 : 1000 lifetime risk of second cancer/leukaemia over the subsequent 40 years (Dalal *et al*, 2006). A chest X-ray is a low-cost low-risk procedure, which is likely to pick up nodules of 1 cm or greater in the lungs, pleurally based lesions or mediastinal masses (Dalal *et al*, 2006).

### General principles

Throughout these recommendations, we have considered the following data on imaging. The chest can be imaged both by chest X-ray and CT of the chest but thoracic relapse in nonseminomatous (NS) will for the most part be marker positive, and the great majority of GCTs that relapse will have significant disease burden outside the chest (Gietema *et al*, 2002; Oldenburg *et al*, 2006; Martin *et al*, 2007). Computerised tomography chest may pick up small marker-negative lesions not visible on chest X-ray, but unless the detection of such small lesions is likely to have prognostic significance, we omit chest CT from our follow-up schedules due to the significant additional radiation exposure. Although one study suggests no additional benefit to using chest X-ray, we feel that if CT of the chest is omitted it is worth performing a chest X-ray (Gietema *et al*, 2002). While pelvic CT forms an integral part of initial staging, there is minimal value in routinely scanning the pelvis as part of the follow-up. While there is no firm evidence base, there is a perceived increased risk of pelvic lymph node disease following scrotal interference, and, for this reason, we include a pelvic CT in this setting (White *et al*, 1997).

For each clinical scenario, we have reviewed the frequency and time to relapse both from the published literature and our own experience, and defined the frequency and nature of the follow-up according to principles previously outlined (Appendix A). We have graded the evidence and recommendations according to the guidelines set out by the American Society of Clinical Oncology (Appendix B) and these are given in square brackets. Recommendations without grading were based on the extensive clinical experience of clinicians from the Royal Marsden Hospital.

## SEMINOMA

### Stage I

Approximately, 75% of men who are diagnosed with seminoma have stage I disease, for which management options following orchidectomy consist of surveillance or adjuvant treatment in the form of either radiotherapy or, more recently, a single cycle of carboplatin chemotherapy (Oliver *et al*, 2005). The disease-specific survival for stage I disease approaches 99% independent of the management strategy used (Oldenburg *et al*, 2006).

#### Seminoma stage I surveillance

Eighty per cent of patients with stage I seminoma will be cured with orchidectomy alone, suggesting that only 20% of patients might benefit from adjuvant treatment. The identification of tumour size greater than 4 cm and rete-testis invasion as prognostic factors, has allowed further risk stratification of this group of patients. If neither risk factor is present, then the risk of relapse is less than 12%, this increases to 15% if one factor is present and over 30% if both are present (Warde *et al*, 2002). We consider patients with none or one risk factor as ideal candidates for surveillance, and, in those with both risk factors, we feel that there is a stronger argument for adjuvant treatment. However, as patients who relapse on surveillance are routinely cured with subsequent treatment, surveillance can be considered as an option for all patients with stage I disease. The overall crude relapse rate for patients managed with surveillance is 15.2–19.3% (Chung *et al*, 2002; Warde *et al*, 2002; Choo *et al*, 2005; Oldenburg *et al*, 2006). Most relapses occur in the first 2 years with a >5% annual hazard rate (AHR); relapses after 2 years are rare but are reported to occur up to 6 years after initial diagnosis (Warde *et al*, 2002; Oldenburg *et al*, 2006). The majority of relapses are in the para-aortic nodes, followed by mediastinal, supraclavicular nodes and lung metastasis (Dieckmann *et al*, 2005; Oldenburg *et al*, 2006). Note that only 30% of seminoma relapses will be marker positive. No studies have addressed the optimal scanning or follow-up frequency with widely differing policies reported (Huddart and Joffe, 2006). We suggest 3 monthly clinic visits and markers with 6 monthly CT scans of the abdomen and a chest X-ray for the first 2 years, based on need for regular cross-sectional imaging in the highest risk period [III, B]. The pelvis should only be scanned if there has been scrotal interference or previous pelvic surgery [III, B]. Follow-up should reduce to 4 monthly in year 3 and then 6 monthly until year 5. Computerised tomography scans and chest X-rays are performed annually until year 5 [III, B]. Annual follow-up with clinical examination and markers should continue for a further 5 years prior to discharge [III, B].

#### Seminoma stage I adjuvant radiotherapy follow-up

Historically, adjuvant treatment has taken the form of radiotherapy resulting in cure rates of 97–100%. Following two MRC trials investigating field and dose (Fossa *et al*, 1999a; Jones *et al*, 2005), the current European recommended schedule for adjuvant radiotherapy is 20 Gy in 10 fractions to the para-aortic nodes. Relapses in the treatment field are extremely uncommon; however, when the pelvic field is omitted, there is a small but statistically higher rate of pelvic relapse (Fossa *et al*, 1999a). In a combined review of 1535 patients treated with para-aortic radiotherapy, the relapse rate at 5 years was 3.6%, with 0.3% of relapses occurring in the abdomen (Martin *et al*, 2007). Most relapses occur in the first 2 years. The annual hazard ratio from the largest reported series for relapse between 2 to 6 years is 0.25–1% (Martin *et al*, 2007). For patients treated with para-aortic radiotherapy, we suggest 3 monthly clinical examination and markers for the first year and 4 monthly for the second year, and then 6 monthly until 5 years [III, B]. Computerised tomography scans of the pelvis only (unless a clinical reason exists to scan abdomen) performed annually for the first 2 years and then at 5 years prior to discharge [IV, C]. The value of pelvic CT is unclear. One publication on omitting pelvic CT reported satisfactory overall outcomes, but a number of patients presented with large symptomatic pelvic masses (Livsey *et al*, 2001). A chest X-ray should be performed at 3 and 6 months and then annually until discharge. The extremely low risk of relapse after 5 years (approaching 0%), makes follow-up beyond 5 years unnecessary (Shahidi *et al*, 2002) [III, B].

#### Seminoma stage I follow-up after single-agent carboplatin

An alternative adjuvant treatment to radiotherapy is a single cycle of carboplatin chemotherapy. The use of a single cycle of carboplatin has been investigated by a number of groups (Dieckmann *et al*, 2000; Oliver *et al*, 2005). Most notably, Oliver *et al* reported a randomised trial of 1477 patients demonstrating no inferiority of a single cycle of carboplatin as compared with para-aortic radiotherapy with respect to disease-free and overall survival. The trial has only reported 4-year follow-up data and although there are limited data on risk of late relapse and on long-term toxicity, in many UK centres the use of carboplatin for stage I seminoma now exceeds the use of radiotherapy. Five-year relapse rates for a single cycle of carboplatin are 6.1% with more than 80% of relapses occurring in the abdomen (Martin *et al*, 2007) (in contrast to patients treated with para-aortic radiotherapy where abdominal relapses are extremely uncommon). We, therefore, recommend CT scan of the abdomen and a chest X-ray yearly, for the first 2 years and again at 5 years; the pelvis should only be scanned if there has been scrotal interference or previous pelvic surgery [III, B]. Patients should have a clinical examination and markers at 1 month after chemotherapy, and then 3 monthly for the first year, 4 monthly for the second year and then 6 monthly until year 5. Data on late relapses in the carboplatin-treated patients are not yet available, and, for this reason, it is recommended that these patients should also be followed for 10 years.

### Stages IIa/b

Stage IIa seminoma includes patients with para-aortic lymph nodes up to 2 cm in size. Stage IIb disease includes those with nodes 2–5 cm. There is considerable variability on how stage IIa/b seminomas are treated. The treatment options for stage IIa/b seminomas include, para-aortic and iliac node radiotherapy (Schmidberger *et al*, 1997; Warde *et al*, 1998; Classen *et al*, 2003; Chung *et al*, 2004), three cycles of BEP (bleomycin, etoposide and cisplatin) or four cycles of EP (etoposide and cisplatin) chemotherapy or a combination of carboplatin chemotherapy and para-aortic radiotherapy (Warde *et al*, 1998; Arranz Arija *et al*, 2001; Patterson *et al*, 2001). All three of the above options provide high rates of cure, but with differing toxicity profiles. Patients treated with chemotherapy alone should be followed according to Stage IIc–IV Guidelines, but we present our protocol for follow-up post-combination carboplatin radiotherapy.

#### Stage IIa/b (post chemoradiotherapy) follow-up

Data obtained from the trials of a single cycle of carboplatin in stage I seminoma suggest that this treatment is sufficient to eradicate microscopic disease in the great majority of patients. We know that radiotherapy is highly effective in eradicating disease with up to 5 cm in size in the para-aortic nodes (Classen *et al*, 2003; Chung *et al*, 2004). Our current protocol for treating patients with stage IIa/b disease, is to give one cycle of carboplatin (AUC 7) followed 4 weeks later by para-aortic radiotherapy to a dose of 30 Gy in 15 fractions based on 5-year relapse-free survival probability of 96.9% (Patterson *et al*, 2001) achieved at our institution. We suggest 3-monthly clinic examinations and markers for the first year, 4 monthly for the second year, 6 monthly until year 5 and then annual review. Computerised tomography scan of the pelvis and chest X-ray should be done at 3 months and again at 1, 2 and 5 years [III, B]. The follow-up schedule is specific to this protocol.

### Stages IIc–IV

There is general agreement that best treatment for stage IIc–IV seminomas involves multiagent platinum-containing chemotherapy (usually BEP). There is evidence from a large prospective randomised trial that three cycles of BEP is equivalent to four cycles for good prognosis GCTs (de Wit *et al*, 2001).

#### Seminoma stage IIc+ follow-up

With large volume seminoma, it is not uncommon for there to be a residual mass following chemotherapy. In most cases, this represents fibrotic scar tissue. Attempted surgical removal can be difficult. In most cases, the masses will resolve in time, but if larger than 3 cm the chance of active malignancy is higher (Puc *et al*, 1996). If PET scanning is available, then data from De Santis *et al* (2004) suggests that 80% of patients with active disease can be identified and should be undertaken for masses >3 cm. Following PET, we suggest performing CT scans at regular intervals (6 monthly to annually) until complete response or a stable calcified mass is the only residuum. When this is achieved, the low rate of relapse makes further scanning unnecessary [III, B]. The follow-up schedule for advanced seminoma is the same as for NSGCT, but patients can be discharged after 5 years if free of relapse (Shahidi *et al*, 2002).

## NONSEMINOMA

### Stage I

The options for men with stage I NSGCTs (post orchidectomy) are close surveillance or adjuvant chemotherapy (in the form of two cycles of BEP chemotherapy). Adjuvant primary retroperitoneal lymph node dissection (RPLND) is rarely performed within the United Kingdom, but we present a discussion of the pertinent issues.

#### NS stage I surveillance

Retrospective series identified the histological presence of vascular invasion as the strongest predictor of relapse in stage I NSGCTs (Freedman *et al*, 1987; Vergouwe *et al*, 2003). Surveillance as a strategy for the management of stage I tumours was prospectively studied by the Medical Research Council and their results were published in 1992 (Read *et al*, 1992). In the absence of high-risk features where relapse rates approach 50%, among the usual population undergoing surveillance a relapse rate of around 20% is to be expected. Surveillance protocols should, therefore, be directed at promptly detecting these relapses, as with treatment at this stage disease-free survival rates of 98% should be achieved.

It is clear from a number of series that the vast majority of relapses will occur within 2 years, most in the first year of surveillance. In the original prospective study, 80% (of 100 relapses) occurred within the first year (54 of these in the first 6 months) and 92% by 2 years (Read *et al*, 1992). A number of subsequent studies have demonstrated similar results and are summarised in Table 1. A series of 478 patients (stage I NSGCT) undergoing surveillance at the Royal Marsden Hospitals (Royal Marsden, unpublished data) with a relapse rate of 24% showed almost identical results with 80% of relapses occurring within 1 year, 90% within 2 years, 94% by 3 years and 97% by 4 years. The subsequent relapses occurred at 6 years (one patient) and 7 years (two patients).

The site and subsequent detection of relapse was assessed in our Royal Marsden series. 72/111 (67%) were marker positive and in 58 (51%) this was the flag. In 15 (14%) this was the only detectable abnormality. Sixty-two patients (56%) had evidence of retroperitoneal adenopathy, and in 55 (50%) this was the only anatomical site of relapse. Of these, 18 (16%) were marker negative, making cross-sectional abdominal imaging mandatory in surveillance protocols. Thoracic relapse rates of around 20% can be expected but methods of detection remain controversial, predominantly as drawing conclusions from small series is unreliable. From a series of 168 patients, 8/42 relapses (19%) occurred within the chest (Harvey *et al*, 2002), although 7 had other indicators of relapse (raised markers and abnormal abdominopelvic CT scans) and all 8 had abnormal chest radiographs, and the authors concluded that there is no requirement for thoracic CT. Some may go further – of 170 patients undergoing surveillance for stage I NS, all recurrences were detected with clinical history and examination, markers and abdominopelvic CT, and it was felt that the omission of chest radiographs would not have altered the outcome (Sharir *et al*, 1999). However, of the 111 Royal Marsden patients who experienced relapse, there were 8 (7%) in whom CT scans of the thorax, performed on the surveillance protocol, were the only detector of relapse.

Our suggested protocols then focus on the first year with investigations reducing in intensity in subsequent years. The patients have markers checked monthly for the first year, with 2-monthly chest X-rays and clinical examination [III, B] and CT scans (abdomen only unless pelvis deemed high risk) at 3 months and 1 year [I, A]. This is broadly similar to the recent NCCN Guidelines, although reduces the frequency of CT, as supported by data from the recently presented MRC TE08 trial, where a two-scan strategy was no less effective than performing five scans (Kondagunta *et al*, 2003; Rustin *et al*, 2007). From the above data, it is clear that relapse after 5 years is so infrequent as to merit discharge at this point. Given the increasing awareness of long-term side effects, we also advocate assessing cardiovascular risk factors in these untreated control patients at 2 and 5 years [III, B].

#### NS stage I post-adjuvant chemotherapy follow-up

Patients in whom the rate of relapse is expected to approach 50% (ie, possessing high-risk features, predominately vascular invasion) are also candidates for surveillance as treatment at relapse confers identical long-term survival outcomes, but may also be offered adjuvant chemotherapy, most commonly in the form of two cycles of BEP. This has been shown to reduce the rate of relapse to <5% (Cullen *et al*, 1996). The proven GCT relapses seen in this trial occurred after 7 months and were detected by raised markers, and a published review including 148 patients treated with adjuvant chemotherapy demonstrated six (4%) relapses, all within 2 years of treatment (Oliver *et al*, 2004).

Reflecting both this much reduced rate of relapse and the conserved tendency to early presentation of recurrence, our protocol reduces the frequency of investigation and follow-up visit at an earlier stage than the previously described for surveillance [III, B]. We suggest one scan of abdomen be performed at 6 months as occasionally a microscopically involved lymph node may increase in size due to the presence of teratoma differentiated [V, D]. As treatment has been administered, assessment of late effects is increasingly important, our protocols assess this at 2 and 5 years post treatment [III, B], but lifelong awareness of the risk of treatment-related effects is important.

#### NS stage I post-adjuvant retroperitoneal lymph node dissection follow-up

Adjuvant RPLND allows more accurate histopathological staging of patients with around 30% of patients deemed stage I by conventional radiological and marker evaluation having pathological evidence of nodal metastases (ie, stage II) at surgery. Such patients are typically offered ‘adjuvant’ chemotherapy in the form of two cycles of BEP with subsequent recurrences uncommon indeed (Donohue *et al*, 1993; Spermon *et al*, 2002; Albers *et al*, 2003). Patients without evidence of nodal metastases demonstrate relapse rates between 10 and 13%, the vast majority of which are pulmonary in nature and occur within the first year.

We would advocate following up patients treated with RPLND and two cycles of BEP (ie, node positive) as post-adjuvant chemotherapy (above). Around 30% node-positive patients not receiving further treatment relapse (Donohue *et al*, 1993) and as such should be followed up along the NS stage I surveillance protocol, as should node-negative patients, although abdominal relapse post-RPLND should be uncommon.

### Stage II–IV NSGCT

Germ cell tumours are one of the rare instances where the majority of patients with disseminated disease can be cured, predominately with combination chemotherapy, although often requiring surgery (and less frequently radiotherapy) to eradicate residual disease. Overall, around 80% should achieve complete clinical +/− radiological response to treatment (depending upon IGCCCG prognostic factors) (Mead *et al*, 1992; Sonneveld *et al*, 2001; Horwich *et al*, 2006). Radiological assessment of response continues until residual disease has either been surgically resected or completely resolved, with a CT scan performed at this point as a baseline for follow-up.

#### Disseminated disease (NS/seminoma), post-curative treatment follow-up

Once curative treatment (CR) has been achieved and treatment completed, relapse rates of up to 10% might be expected. The majority of these will occur within the first year, although a late relapse rate (that occurring >2 years post completion of treatment) of around 3% is observed (Gietema *et al*, 2002; Ronnen *et al*, 2005). Of 353 patients treated in the Netherlands between 1977 and 1999, 290 (82%) achieved CR following cisplatin-based chemotherapy and surgery as required. Thirty-three of these (11%) subsequently relapsed, with a median time to relapse of 17 months (range: 6–179). In 27 (81%) of these, elevated markers ‘flagged’ the relapse, and a further 4 markers corresponded to recurrence found at physical examination. Two patients with brain metastasis presented with neurological symptoms. In no patients did the chest radiograph assist with detection of recurrence, and the authors point out that following ESMO Guidelines would have resulted in over 6000 X-rays in total for this period (Gietema *et al*, 1992).

Specifically regarding late recurrences (Ronnen *et al*, 2005), 17/551 patients relapsed after at least 2 years post chemotherapy, with a median time to relapse of 7.8 years (range: 2.7–18.7) and 9/17 presenting with clinical symptoms of recurrence. In all, 3 out of the 17 presented with raised markers (although a further 9 subsequently demonstrated raised markers), and, in this series, two relapses were detected by abnormal chest X-ray. From a retrospective review of 1263 patients with TGCT treated at the Royal Marsden Hospital (513 with metastatic NSGCT), 53 had recurrences after 2 years, 14 of which occurred later than 5 years (9/14 detected at routine annual follow-up and 5 presenting with interval symptoms). Twelve of these very late recurrences were in patients treated for metastatic NS (giving an annual risk of recurrence in these patients of 1% between 5 and 10 years) (Shahidi *et al*, 2002).

Our protocols therefore utilise less imaging, although in the absence of randomised data we continue to recommend 4-monthly chest X-rays for the first 2 years, but annually after that [III, B]. Markers are checked 2 monthly for the first year, 4 monthly for the second and 6 monthly after that [III, B] for up to 5 years. The continued risk of late relapse in NS patients means that we recommend continuing to follow up these patients annually from 5 to 10 years and every 2 years subsequently [V, D]. Late relapse is rare (Shahidi *et al*, 2002) in metastatic seminoma so would routinely discharge seminoma patients at 5 years [III, B].

## ASSESSING LATE TOXICITY

Although a thorough discussion regarding long-term side effects of treatment is beyond the scope of this article, a growing body of data demonstrates that survivors of TGCTs are at increased risk of hypogonadism, and a metabolic syndrome including obesity, hypertension, diabetes and hypercholesterolaemia (Vaughn *et al*, 2002). We, therefore, include in our protocols assessing patients for these features patients at 2, 5 and 10 years. We would assess BP, glucose, fasting cholesterol, LH and testosterone at these time points. Patients should be advised not to smoke and to be counselled regarding weight gain. A history of cardiac disease or other health problems should be sought particularly beyond 5 years of follow-up. It is not known if other screening tests for coronary artery disease or for secondary cancer would be advantageous, but patients should be encouraged to avail themselves of any relevant national screening programmes such as the one being developed for colorectal cancer.

## CONCLUSIONS

Managing patients with TGCTs is a complex business requiring multidisciplinary teamwork and multiple clinical scenarios. While evidence for best practice in terms of treatment is widely available and continuously evolving, the important issues regarding follow-up schedule are for the most part left to individual preference. The standardising of follow-up would result in optimising risk/benefit ratios for individual patients, while ensuring economic use of resources. Furthermore, this should enable future trials to address specific issues around follow-up giving meaningful and useful results.

These guidelines have been adopted by the South East England Testicular Cancer supraregional network.

## Figures and Tables

**Table 1:** Time course of relapse in surveillance of stage I nonseminoma

			**Cumulative relapses**		
**Reference**	**Patients**	**Relapses**	**Year 1 (%)**	**Year 2(%)**	**Year 3(%)**
MRC, Read *et al* (1992)	373	100 (27%)	80	92	100
Atsu *et al* (2003)	132	32 (24%)	87	100	100
Daugaard *et al* (2003)	301	86 (29%)	80	89	95
Divrik *et al* (2006)	211	66 (31%)	79	95	—
Drury A Royal Marsden, unpublished)	478	115 (24%)	80	90	97

## APPENDIX B

### Levels of evidence and grading of recommendation

**Level**: **Type of evidence**
I: Evidence is obtained from meta-analysis of multiple, well-designed, controlled studies. Randomised trials with low false-positive and low false-negative errors (high power).
II: Evidence is obtained from at least one well-designed experimental study. Randomised trials with high false-positive and/or negative errors (low power).
III: Evidence is obtained from well-designed, quasi-experimental studies such as nonrandomised, controlled single-group, pre–post, cohort, time or matched case–control series.
IV: Evidence is from well-designed, nonexperimental studies such as comparative and correlational descriptive and case studies.
V: Evidence is from case reports and clinical examples.
**Grade**: **Grading of recommendation**
A: There is evidence of type I or consistent findings from multiple studies of types II, III or IV.
B: There is evidence of types II, III or IV and findings are generally consistent.
C: There is evidence of types II, III or IV but findings are inconsistent.
D: There is little or no systematic empirical evidence.
